# Osthole enhances the bone mass of senile osteoporosis and stimulates the expression of osteoprotegerin by activating β-catenin signaling

**DOI:** 10.1186/s13287-021-02228-6

**Published:** 2021-02-27

**Authors:** Zhen-Xiong Jin, Xin-Yuan Liao, Wei-Wei Da, Yong-Jian Zhao, Xiao-Feng Li, De-Zhi Tang

**Affiliations:** 1 Longhua Hospital, Shanghai University of Traditional Chinese Medicine, Shanghai, 200032 China; 2 Institute of Spine, Shanghai University of Traditional Chinese Medicine, Shanghai, 200032 China; 3 Spine Center, Department of Orthopaedics, Shanghai Changzheng Hospital, Navy Medical University, Shanghai, 201705 China

**Keywords:** Osthole, β-Catenin, Osteoprotegerin, Osteoclast, Osteoporosis

## Abstract

**Introduction:**

Osthole has a potential therapeutic application for anti-osteoporosis. The present study verified whether osthole downregulates osteoclastogenesis via targeting OPG.

**Methods:**

In vivo, 12-month-old male mice were utilized to evaluate the effect of osthole on bone mass. In vitro, bone marrow stem cells (BMSCs) were isolated and extracted from 3-month-old OPG^−/−^ mice and the littermates of OPG^+/+^ mice. Calvaria osteoblasts were extracted from 3-day-old C57BL/6J mice or 3-day-old OPG^−/−^ mice and the littermates of OPG^+/+^ mice.

**Results:**

Osthole significantly increased the gene and protein levels of OPG in primary BMSCs in a dose-dependent manner. The deletion of the *OPG* gene did not affect β-catenin expression. The deletion of the β-catenin gene inhibited OPG expression in BMSCs, indicating that osthole stimulates the expression of OPG via activation of β-catenin signaling.

**Conclusion:**

Osthole attenuates osteoclast formation by stimulating the activation of β-catenin-OPG signaling and could be a potential drug for the senile osteoporosis.

## Summary

Osthole has the potential therapeutic applications for anti-osteoporosis. The current study suggested that osthole attenuates osteoclast formation by stimulating the activation of β-catenin osteoprotegerin (OPG) signaling and inhibits bone resorption.

## Introduction

Osteoporosis is a systematic skeletal disease that thins and weakens the bones to the point that they become fragile and break easily. It is one of the most disabling consequences of aging [1, 2]. Hip fractures and vertebral fractures are strongly associated with reduction in bone mineral density (BMD) and have been considered the prototypical osteoporotic fractures [3]. In 2010, about 2.7 million hip fractures occurred worldwide, and about half of these (51%) were considered preventable [4, 5]. However, the incidence of all other fractures (non-hip, non-vertebral) is marked, and collectively these fractures result in large economic costs for the population [6]. Fractures show symptoms of pain and an inability to bear weight and almost always require surgical fixation [7]. In addition, the patient’s functional status and quality of life are reduced, with a high risk for short-term mortality, as well as large medical expenses [8, 9].

Estrogen replacement therapy is effective in increasing osteoblast activity but also increases the incidence of breast and uterine cancer [10, 11]. Phytoestrogens have attracted attention due to the potential impacts on the prevention and treatment of osteoporosis. Osthole, a coumarin derivative extracted from *Cnidium monnieri* and Angelica of Chinese herbal medicine, has an estrogenic effect in preventing bone loss in ovariectomized rats [12, 13]. Several studies have confirmed a wide range of pharmacological activities of osthole in humans, such as anti-cancer activity and antihypertensive, anti-arrhythmic, anti-inflammatory, and anti-infection properties; it also promotes osteoblast differentiation [14–16].

Bone marrow stem cells (BMSCs) differentiate into osteogenic, fat, cartilage, and nerve-like cells, with robust in vitro expansion capacity, as well as the potential for multi-directional differentiation [17, 18]. The underlying mechanisms have been studied using recent technological advances in cell labeling and tracing [19, 20]. Subsequently, the lineage tracing methods proposed that bone marrow cells expressing myxovirus resistance-1 (Mx1) harbor the characteristics of BMSCs. The Mx1 protein can restrict several viruses independent of the expression of other interferon (IFN)-induced genes. These cells respond to tissue stress and migrate to the sites of injury, supplying new osteoblasts during fracture healing [21]. Leptin receptor (LepR) is also another marker for identifying BMSCs. LepR-positive cells have been shown to produce osteoblasts and adipocytes in bone marrow [22]. In addition, cells expressing gremlin-1 have been isolated from bone marrow; these cells are capable of bone formation rather than adipogenesis [23]. Thus, BMSCs are widely used in clinical research at the cellular level of bone and cartilage tissues, including cartilage repair.

Our previous study demonstrated that osthole significantly stimulated osteoblast differentiation and bone formation by the activation of Wnt/β-catenin-Bmp2 signaling [24]. The previous meta-analysis showed that BMSCs promote bone cell maturation, ossification, and restore bone mechanical properties in osteoporotic fractures [25]. Although osthole can promote bone formation, its effect on bone resorption and the underlying mechanism is yet to be elucidated. In this study, we performed the in vivo and in vitro experiments to examine the effect of osthole on osteoclast formation.

## Materials and methods

### Mice and reagents

All animal protocols were approved by the Institutional Review Board of Longhua Hospital, Shanghai University of Traditional Chinese Medicine (China). C57BL/6J wild-type mice were purchased from the Institute of Zoology, Chinese Academy of Sciences. Osteoprotegerin (OPG) knockout (KO) mice, and OPG wild-type (WT) mice were purchased from the Shanghai Bio model Organism Science and Technology Development Co., Ltd. (China). Osthole, with 98% purity, was purchased from the Shanghai Institute for Drug and Quarantine Bureau. The reagents used in the experiment are listed in Table S1.

### Animal study

Six-month-old C57BL/6 mice, specific pathogen-free (SPF), were purchased from the Institute of Zoology, Chinese Academy of Sciences. Four months later, C57BL/6 mice of 1 month old were purchased again, and the experiment was performed when the mice were 3 and 12 months old. Next, the 12-month-old male mice were randomized equally (*n* = 6) into two groups: treatment and control group. The treatment group was intervened with osthole (5 mg/kg/day) by intraperitoneal injection once a day for 4 weeks, and the control group was intervened with vehicle (corn oil) by intraperitoneal injection once a day for 4 weeks. After sacrifice, the lumbar vertebrae were harvested for evaluation.

### Microcomputed tomography (μCT) analysis

The fourth lumbar vertebra (L4) was scanned at 18-μm voxel size using the μCT scanner (μCT80, Scanco Medical AG, Bassersdorf, Switzerland). The trabecular bone under the growth plate was segmented using a contouring tool, and the contours were morphed automatically to segment the trabecular bone on all the 100 slices. The three-dimensional (3D) images were reconstructed and analyzed using the software of the μCT system.

### Histological and histomorphometric assays

The fifth lumbar vertebra (L5) was fixed in 4% paraformaldehyde, decalcified, dehydrated, cleared with dimethyl benzene, and embedded in paraffin. At least three consecutive 7-μm sections were obtained from the coronal planes and subjected to tartrate resistant acid phosphatase (TRAP) staining to identify osteoclasts. The histomorphometric assay was performed to determine the number of osteoclasts and the proportion of osteoclast surface using an image auto-analysis system (Olympus BX50; Japan).

### Immunohistochemical staining

The paraffin sections of L3 were deparaffinized by immersing the tissue in xylene, fixing with 4% paraformaldehyde for 15 min, and permeabilizing with 0.5% Triton X-100 for 15 min, followed by fixation with 4% paraformaldehyde for an additional 5 min. Then, the sections were incubated with rabbit anti-OPG monoclonal antibody (1:50) and rabbit anti-β-catenin monoclonal antibody (1:50) at 4 °C overnight and then with horseradish peroxidase (HRP)-conjugated secondary antibody for 30 min. Finally, the slides were mounted and examined using an Image Analysis System (Olympus BX50).

### Cell culture and treatment

Primary BMSCs, extracted from 3-month-old OPG^−/−^ mice and the littermates of OPG^+/+^ mice, were cultured by incubation with macrophage colony-stimulating factor (M-CSF) and receptor activator of nuclear factor (NF)-κB-ligand (RANKL) for 1 week, and then treated with various doses (0.5–100 μM) of osthole for 48 h. Transgenic mice were presented by Professor Chen, Department of Orthopedics, University of Rochester. Primary calvaria osteoblasts, extracted from 3-day-old C57BL/6J mice or 3-day-old OPG^−/−^ mice and the littermates of OPG^+/+^ mice.

### TRAP staining

Primary BMSCs were seeded in a 96-well plate at a density of 3 × 10^5^/mL and treated with M-CSF (44 ng/mL) and RANKL (100 ng/mL) in the presence or absence of osthole (100 μM). The medium was changed every 3 days. After 7-day incubation, the cells were fixed and subjected to TRAP staining to calculate the number of multinuclear (≥ 3 nuclei) osteoclasts.

### Real-time quantitative polymerase chain reaction (qPCR) analysis

Primary calvaria osteoblasts, extracted from 3-day-old C57BL/6J mice, were seeded in 6-well plates at a density of 1 × 10^6^ cells/well. After 2-day culture, the cells were treated with various doses of osthole (1–100 μM) or vehicle for 48 h. Total cellular mRNA was isolated using the RNeasy Mini Kit (Qiagen Corporation, Valencia, CA). An equivalent of 1 μg of total RNA was reverse-transcribed into cDNA using the iScript cDNA synthesis kit (Bio-Rad Laboratories, Inc., Hercules, CA). qPCR analysis was carried out using Absolute QPCR SYBR Green Master Mix in a total volume of 20 μL reaction containing 1 μL of the diluted (1:5) reverse transcription product in the presence of sense and antisense primers of target genes listed as Table 1. *β-actin* served as the internal reference gene. The reaction was as follows: polymerase activation at 95 °C for 15 min, followed by 45 cycles of 95 °C for 20 s, 58 °C for 20 s, and 72 °C for 30 s. All reactions were performed in triplicate.

**Table 1:** Mouse primers for real-time quantitative PCR assays

Genes	Forward primer	Reverse primer
β-actin	5′-TGTTACCAACTGGGACGACGACA-3′	5′-CTGGGTCATCTTTTCACGGT-3′
OPG	5′-CCACTCTTATACGGACAGCT-3′	5′-TCTCGGCATTCACTTTGGTC-3′

### Western blotting analysis

Primary calvaria osteoblasts, isolated from 3-day-old OPG^−/−^ homozygous mice and the littermates of OPG^+/+^ mice, were seeded in 6-well plates at a density of 1 × 10^6^ cells/well. Cells were treated with osthole (100 μM) or vehicle for 48 h and cell lysates were extracted using protein extraction reagents (PER) (Thermo Scientific, Waltham, MA, USA). The protein concentration is 10 μg, and by Sodium Dodecyl Sulfonate-Polyacrylamide gel electrophoresis (SDS-PAGE) and the 10% of gel. Proteins were transferred onto a PVDF membrane (Bio-Rad, Hercules, CA, USA). Subsequently, the membrane was blocked with 5% non-fat milk in phosphate-buffered saline and Tween-20 (PBST) for 1 h at room temperature and probed with primary antibodies overnight at 4 °C, followed by HRP-conjugated secondary antibodies (Thermo Scientific) for 1 h at room temperature. The intensities of the immunoreactive bands were detected using a SuperSignal West Femto Maximum Sensitivity Substrate Kit (Thermo Scientific). Rat anti-OPG and rabbit anti-β-catenin monoclonal antibodies were the primary antibodies, and mouse anti-β-actin monoclonal antibody was used as an internal reference.

### In vitro deletion of the *β*-*catenin* gene

The in vitro deletion of the *β-catenin* gene was performed as described previously [24]. calvaria osteoblasts isolated from 3-day-old *β-catenin*^*fx/fx*^ mice were seeded in 6-well culture plates at a density of 1 × 10^6^/well and cultured for 6 days. Then, the cells were infected with Ad-GFP or Ad-Cre (titer, 4 × 10^8^ pfu/mL; Baylor College of Medicine, Houston, TX, USA) for 72 h, and Ad-GFP was used as a control to monitor infection efficiency. After recovery for 48 h, cells were treated with or without osthole (100 μM) for 48 h. Real-time qPCR assay was performed to examine the expression of *β-catenin* and *OPG*. All reactions were performed in triplicate.

### Statistical analysis

All experiments were conducted independently at least three times. The data were expressed as mean ± standard deviation (SD) and analyzed using SPSS 24.0 software and GraphPad Prism 8. The statistically significant differences were analyzed using Student’s *t* test of variance. We compared 3-month-old mice vs. 12-month-old mice and osthole vs. vehicle. ImageJ software was employed to quantitate the grayscale intensities. *P < 0.05 indicated statistically significant difference.

## Results

### Bone loss was severed in 12-month-old mice

**Fig. 1:**
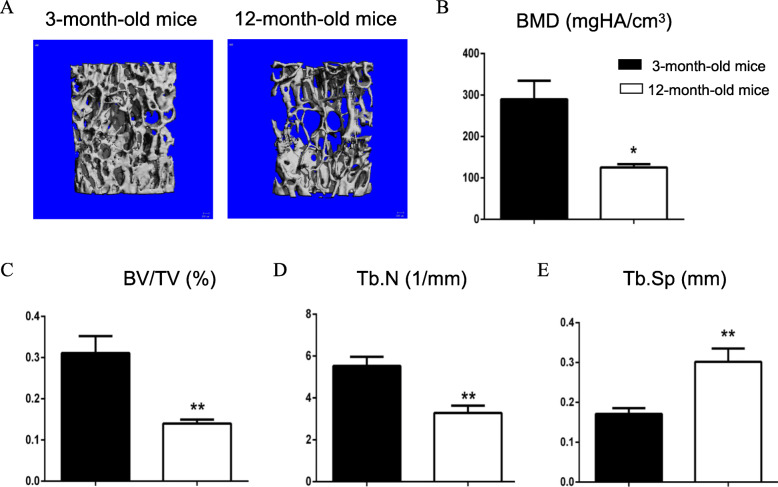
Bone mass was significantly reduced in 12-month-old mice. Three-month-old and 12-month-old C57BL/6 mice were used to evaluate the bone mass. **a** The μCT 3D image analysis of the fourth lumbar vertebra (L4) demonstrated an apparent bone loss in 12-month-old mice compared to 3-month-old mice. The quantitative analysis showed that bone volume (BV) and bone mineral density (BMD) were significantly decreased in 12-month-old mice compared to 3-month-old mice (**b**, **c**, *n* = 6). *P < 0.05, **P < 0.01, unpaired Student’s *t* test (3-month-old mice vs. 12-month-old mice)

3- and 12-month-old C57BL/6J mice were used to evaluate the bone mass. The μCT 3D image analysis on L4 showed the loss of trabecular bone in 12-month-old mice compared to that in 3-month-old mice (Fig. 1a). The quantitative analysis showed that bone volume/total volume (BV/TV) and bone mineral density (BMD) of aged mice were significantly decreased (*P* < 0.05, Fig. 1b, c) compared to those in young mice, suggesting that aged mice display decreased bone mass.

### Osthole inhibited aging-induced bone loss

**Fig. 2:**
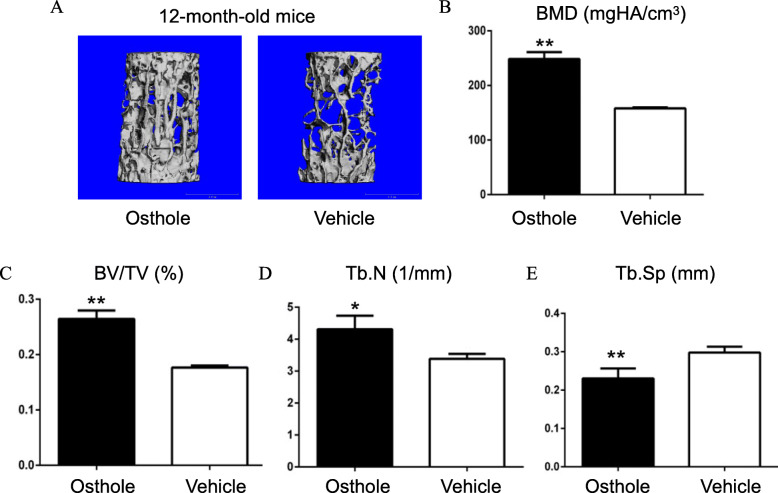
Osthole significantly increased the bone mass of aged mice. Twelve-month-old C57BL/6J mice were treated with osthole (5 mg/kg/day) or vehicle (corn oil) by intraperitoneal injection once a day for 4 weeks. Then, mice were sacrificed, and the lumbar vertebrae were harvested for evaluation. **a** The μCT 3D image analysis of L4 samples showed that osthole significantly increased bone mass in aged mice. **b**, **c** An apparent increase in bone volume (BV) and bone mineral density (BMD) was observed in aged mice after treatment with osthole by the quantitative analysis (*n* = 6). **d**, **e** Osthole was found to significantly increase Tb.N (*P* < 0.05) and decreased Tb.Sp (*P* < 0.05). *P < 0.05, **P < 0.01, unpaired Student’s *t* test (osthole vs. vehicle)

Osthole significantly increased the bone mass of aged mice (Fig. 2a). The quantitative analysis showed an apparent increase in 67% of BMD (*P* < 0.05) and 75% of BV/TV in aged mice (*P* < 0.05) after treatment with osthole (Fig. 2b, c) that significantly increased Tb.N (*P* < 0.05, Fig. 2d) and decreased Tb.Sp (*P* < 0.01, Fig. 2e). These data demonstrated that osthole inhibited aging-induced bone loss.

### Osthole inhibited osteoclast formation in aged mice

TRAP staining was performed on sections of L5. Osthole decreased the TRAP-positive number of multinucleated osteoclasts (Figure S1A, B), and reduced the percentage of osteoclast surface in aged mice post-osthole treatment (Figure S1C). These data showed that osthole inhibits osteoclast formation in aged mice. Conversely, we found that osthole could not inhibit osteoclast formation in *OPG* gene knockout mice (Figure S1D, E), suggesting that the effect of osthole might be effectuated via OPG signaling.

### Osthole inhibited osteoclastogenesis in a dose-dependent and OPG-dependent manner

**Fig. 3:**
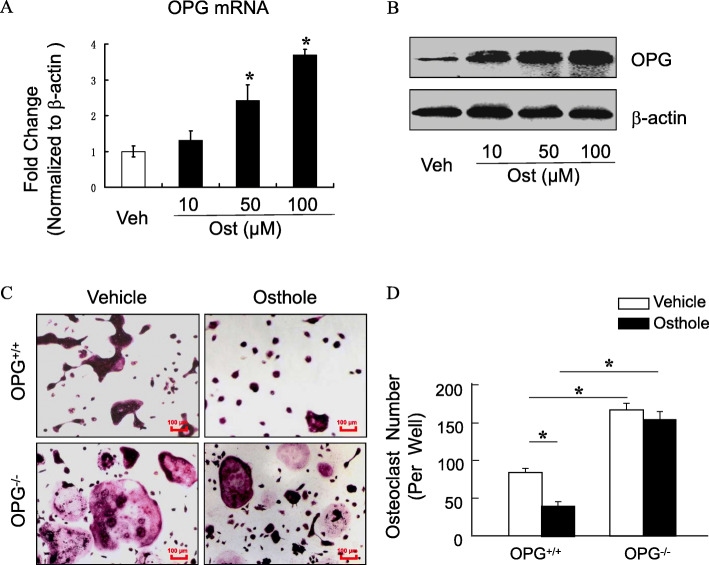
Osthole inhibits osteoclastogenesis in a dose-dependent and OPG-dependent manner. **a** Osteoblasts were cultured and treated with various doses of osthole (10–100 μM) or vehicle (DMSO) for 2 days; OPG expression was detected using real-time PCR assay. **b** The protein expression of OPG was detected using Western blot assay. **a, b** showed that osthole significantly increased the mRNA and protein level of OPG in a dose-dependent manner. **c **BMSCs were isolated from 3-month-old OPG^−/−^ mice and the littermates of OPG^+/+^ mice, TRAP staining was performed. The scar bar is 100 μm. **d **Quantification of osteoclast number for osthole in **c**. The number of multinucleated TRAP-positive cells (> 3 nuclei) in each representative view area at × 40 magnification. *P < 0.05, unpaired Student's *t* test. **c,** **d** Osthole inhibited the formation of osteoclasts in an OPG-dependent manner

OPG/RANKL signaling plays a major role in osteoclast formation. To determine the mechanism of osthole in suppressing osteoclast formation, we examined its effect on the expression of OPG and RANKL. Osthole (10, 50, 100 μM) significantly enhanced the expression of *OPG* mRNA in a dose-dependent manner (*P* < 0.05), and compared with the vehicle group, a dose of 100 μM exerted maximum effect with 3.8-fold increase (Fig. 3a). Next, we found that osthole significantly increased the protein level of OPG in a dose-dependent manner (Fig. 3b). To further determine if osthole-inhibited osteoclastogenesis is OPG dependent, BMSCs were isolated from 3-month-old OPG^−/−^ mice and the littermates of OPG^+/+^ mice, cultured with M-CSF (44 ng/mL) and RANKL (100 ng/mL), plus osthole (100 μM) or vehicle for 7 days; subsequently, TRAP staining was carried out, and the number of osteoclasts was counted. As shown in Fig. 3c and d, osthole significantly inhibited osteoclast formation in OPG^+/+^ mice (*P* < 0.05). Conversely, in OPG^−/−^ mice, the formation of osteoclasts was not affected. These data suggested that osthole inhibits osteoclastogenesis in an OPG-dependent manner.

### Osthole promoted the expression of OPG through activation of β-catenin signaling

**Fig. 4:**
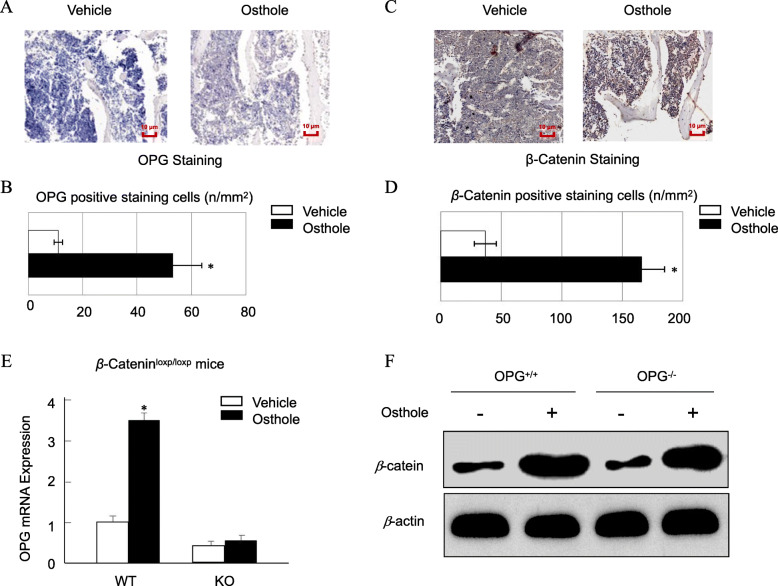
Osthole promotes the expression of OPG through activation of β-catenin signaling. **a** OPG immunostaining was performed in L5 sections of 12-month-old C57BL/6 mice. **b** Osthole increased the OPG protein level in aged mice. **c** β-catenin immunostaining was performed in L5 sections of 12-mont-hold C57BL/6 mice. **d** Osthole increased the β-catenin protein level in aged mice. **e** Osteoblasts were isolated from 3-day-old β-catenin^fx/fx^ mice, infected with Ad-Cre or Ad-GFP, and treated with or without 100 mM osthole. After 2 days, the expression of OPG was detected using real-time PCR assay. *P<0.05. **f** Osteoblasts were isolated from 3-day-old OPG−/− mice and the littermates of OPG^+/+^ mice were treated with osthole (100 μM) or vehicle for 2 days; the expression of β-catenin was detected using the Western blot assay

Our recent studies have demonstrated that OPG expression could be activated by β-catenin signaling, and osthole could activate β-catenin signaling [24, 26, 27]. First, we examined the expression of OPG and β-catenin expression in vivo. The immunostaining data showed that osthole significantly increased the level of OPG (Fig. 4a, b) and β-catenin proteins (Fig. 4c, d) using sections of L5 samples from aged mice. To further confirm if osthole-induced OPG expression is mediated via β-catenin signaling, we performed an in vitro study. Primary BMSCs were isolated from 3-month-old β-catenin^fx/fx^ mice, infected with Ad-Cre or Ad-GFP, and treated with or without 100 μM osthole. After 2 days, the total mRNA was collected, and the expression of *OPG* was detected using real-time PCR assay. We found that the deletion of β-catenin by Ad-Cre infection significantly inhibited osthole-induced expression of OPG (Fig. 4e), while the deletion of OPG did not affect the osthole-induced expression of the β-catenin protein (Fig. 4f). Taken together, these results indicated that osthole promotes the expression of OPG via β-catenin signaling.

## Discussion

**Fig. 5:**
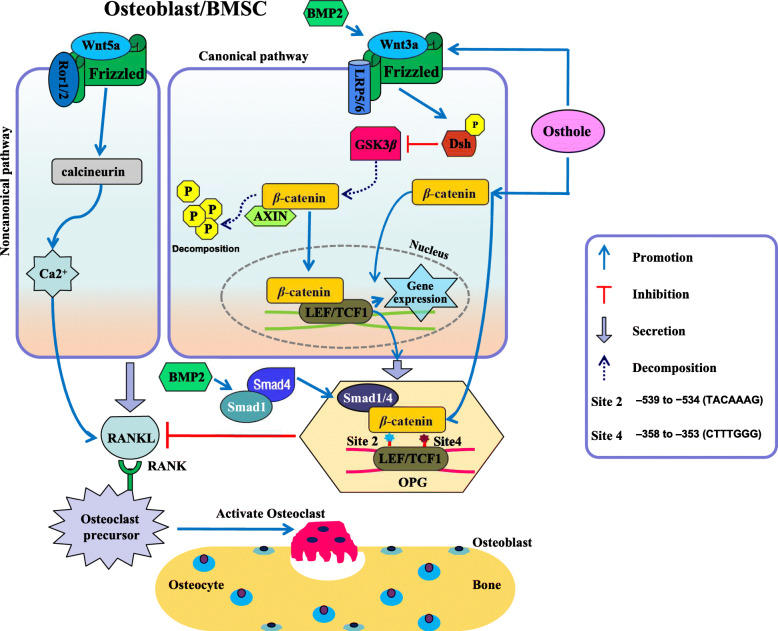
Schematic of the mechanism underlying osthole-mediated inhibition of osteoclast formation and bone resorption

The present study discovered that osthole inhibited bone resorption in aged mice and attenuated osteoclast formation via activated β-catenin-OPG signaling (Fig. 5). Osteoporosis is caused by the disorder of homeostasis between bone formation and bone resorption. Our previous results showed that osthole has the efficacy to promote osteoblastic proliferation and differentiation and act on bone metabolism [24]. Herein, we investigated whether osthole has an impact on the function of osteoclasts. It was firstly used to intervene with the elderly wild-type mice. The μCT analysis showed that the treatment with osthole for 4 weeks significantly increased the bone mass in senile mice. One of the major reasons for the occurrence of osteoporosis is the increased activity and quantity of osteoclasts. Thus, TRAP staining was performed, and the results demonstrated that the number of osteoclasts was decreased in senile mice after intraperitoneal injection with osthole. To further confirm this effect, the dose-dependent effect of osthole on osteoclasts, derived from primary BMSCs after M-CSF and RANKL treatment, was studied. The data showed that osthole decreases the number of osteoclasts in a dose-dependent manner. Next, we performed immunofluorescence staining on bone slices and found that osthole attenuates the activity of functional osteoclasts. Therefore, it could be deduced that osthole inhibits osteoclast formation and osteoclast-involved bone resorptive activity.

Furthermore, we investigated the mechanism of osthole on osteoclast formation. Under normal physiological conditions, the resorption of cartilage and bone is essential for the development and regeneration of the skeleton [28]. Osteoclastogenesis is a complicated process regulated by finely orchestrated interactions between osteoclast precursors and osteoblasts/stromal cells in the bone marrow environment [29, 30]. Osteoblasts produce OPG, a decoy receptor for the receptor activator of RANKL. The binding of RANKL and OPG inhibits the interaction between RANKL and the receptor activator of nuclear factor-kappa B (RANK), a receptor of RANKL; therefore, OPG/RANKL/RANK plays key roles in the process of osteoclastogenesis [31, 32]. The current data showed that osthole significantly promoted the expression of OPG in BMSCs in a dose-dependent manner but did not show an obvious effect on RANKL expression. RANKL is essential for osteoclast formation that is further supported by osteoblasts or their precursors [33]. Recent evidence revealed that osteocytes express high levels of RANKL and contribute to the coupling of bone formation and bone resorption [34, 35]. Herein, we showed that the osthole effects on RANKL could be indirect via overexpression of OPG, which binds with RANKL and inhibits osteoclastogenesis.

In order to demonstrate if osthole-inhibited osteoclastogenesis was OPG-dependent, we performed a rescue experiment using BMSCs from OPG^+/+^ and OPG^−/−^ mice. Osthole decreased the formation of osteoclasts in OPG^+/+^ mice, while it did not affect OPG^−/−^ mice. Therefore, the current results indicated that osthole-attenuated osteoclastogenesis was effectuated via increased expression of OPG in BMSCs. This phenomenon inhibited the binding of RANKL and RANK but did not act on RANKL and RANK directly. The mechanism underlying osthole was further studied with respect to OPG upstream signaling. Reportedly, canonical Wnt pathway upregulates the expression of OPG in osteoblasts and chondrocytes [36, 37]. β-catenin, a key protein of the canonical Wnt pathway, is required to induce the expression of OPG in osteoblasts [38]. However, the inactivation of β-catenin signaling in osteoblasts increased osteoclastogenesis due to insufficient production of OPG [39]. Wnt3a cannot induce the production of normal OPG to inhibit bone resorption while lacking β-catenin in osteoclastic lineage [36, 39]. Cellular and molecular studies have shown that β-catenin banding with TCF proteins regulates the expression of OPG in osteoblasts [40]. In this study, we found that osthole significantly increases the expression of β-catenin and OPG in BMSCs. To further discuss the interaction between β-catenin and OPG, we performed the in vitro rescue experiment using floxed mice (β-catenin) and found that the deletion of the *β-catenin* gene inhibits osthole-induced OPG expression. These results demonstrated that osthole stimulated the expression of OPG through β-catenin signaling.

Canonical Wnt signaling is critical for the differentiation of mesenchymal progenitors into osteoblasts and to assess the connection between osteoblasts and bone metabolism [41]. Released β-catenin might also interact with site 2 (− 539 to − 534 (TACAAAG)) and site 4 (− 358 to − 353 (CTTTGGG)) on the OPG promoter directly, to increase the production and secretion of OPG and inhibit the binding of RANKL and RANK, thereby reducing the formation and activity of osteoclasts [40]. Reportedly, BMP2 also increases the expression of OPG by upregulating Wnt3a expression and promoting the interaction between Samd1/4 and sites 2 and 4 on OPG promoter [42]. On the other hand, Wnt5a activates the noncanonical Wnt signaling, which increases the activity of RANKL to promote osteoclastogenesis [43]. Our previous study showed that osthole significantly stimulates the expression of Wnt3a but not Wnt5a [24].

## Conclusion

Based on the findings, we concluded that osthole inhibits osteoclast formation and bone resorption by stimulating the activation of Wnt3a/β-catenin-OPG signaling (Fig. 5).

## Supplementary Information

**Additional file 1: Table S1.** Reagents List.

**Additional file 2. Figure S1. **OPG and Trap staining.

## Data Availability

The datasets used and/or analyzed during the current study are available from the corresponding author on reasonable request.

## Abbreviations

BMD: Bone mineral density
Mx1: Myxovirus resistance-1
OPG: Osteoprotegerin
μCT: Micro-computed tomography
BMSCs: Bone marrow stem cells
LepR: Leptin receptor
KO: Knockout
WT: Wild-type
DMSO: Dimethyl sulfoxide
qPCR: Quantitative polymerase chain reaction
RANK: Receptor activator of nuclear factor-kappa B
RANKL: The receptor activator of nuclear factor-kappa B ligand
