# Supplementary material for: Osthole enhances the bone mass of senile osteoporosis and stimulates the expression of osteoprotegerin by activating β-catenin signaling
Source: Stem Cell Res Ther. 2021 Feb 27;12:154. doi: 10.1186/s13287-021-02228-6 (PMC7912492; doi:10.1186/s13287-021-02228-6)
Supplement: Supplementary file 1 — Additional file 1: Table S1. Reagents List. [file 13287_2021_2228_MOESM1_ESM.pdf]

Table S1: Reagents List

Name	Company	Address
Recombinant human M-CSF	R&D system	Minneapolis, MN, USA
receptor activator of nuclear factor-kappa B ligand (RANKL) proteins	R&D system	Minneapolis, MN, USA
Dimethyl sulfoxide (DMSO)	Sigma-Aldrich	St. Louis, MO, USA
Fetal bovine serum	Invitrogen Corporation	Carlsbad, CA
DMEM dehydrated medium	Invitrogen Corporation	Carlsbad, CA
penicillin streptomycin combination	Invitrogen Corporation	Carlsbad, CA
RNeasy Mini RNA kit	Qiagen Corporation	Valencia, CA
iScriptcDNA synthesis kit	Bio-Rad Laboratories, Inc	Hercules, CA
PVDF membrane	Bio-Rad Laboratories, Inc	Hercules, CA
Absolute QPCR SYBR Green Master Mix	Thermo Scientific	Waltham, MA
E-PER protein extraction reagents	Thermo Scientific	Waltham, MA
Tartrate-resistant acid phosphatase staining (Trap) kit	Sigma-Aldrich	St. Louis, MO
Mouse bone gla-protein (BGP) ELISA kit	GENTAUR	SanJose, CA
Rabbit anti-OPG monoclonal antibody	Cell Signaling Technology, Inc	Beverly, MA
Rabbit anti-β-catenin monoclonal antibody	Cell Signaling Technology, Inc	Beverly, MA
mouse anti-β-Actin monoclonal antibody	Cell Signaling Technology, Inc	Beverly, MA
Adenovirus-GFP	Baylor College of Medicine	Houston, TX, USA
Adenovirus -Cre	Baylor College of Medicine	Houston, TX, USA
