# Supplementary figures and images for: Osthole enhances the bone mass of senile osteoporosis and stimulates the expression of osteoprotegerin by activating β-catenin signaling
Source: Stem Cell Res Ther. 2021 Feb 27;12:154. doi: 10.1186/s13287-021-02228-6 (PMC7912492; doi:10.1186/s13287-021-02228-6)

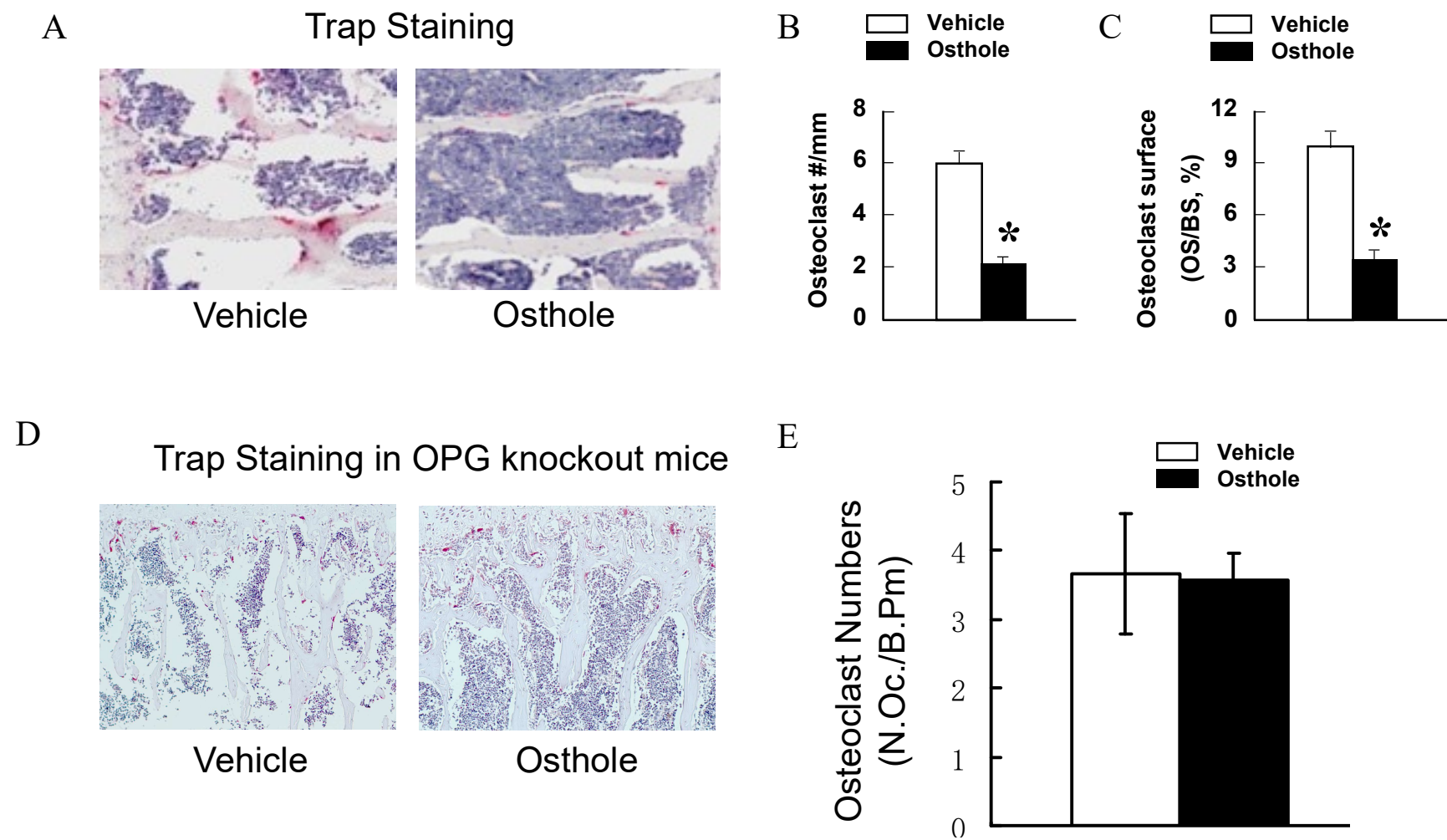

Figure S1

Supplement: Supplementary file 2 — Additional file 2. Figure S1. OPG and Trap staining. [file 13287_2021_2228_MOESM2_ESM.pdf]
